# Uncovering the role of genetic polymorphisms in cervical insufficiency

**DOI:** 10.1002/ijgo.70494

**Published:** 2025-09-04

**Authors:** Kallirhoe Kalinderi, Michail Kalinderis, Maria Chatzidimitriou, Liana Fidani

**Affiliations:** ^1^ Laboratory of Medical Biology‐Genetics, School of Medicine, Faculty of Health Sciences Aristotle University of Thessaloniki Thessaloniki Greece; ^2^ Department of Obstetrics and Gynaecology St George's University Hospital NHS Trust London UK; ^3^ Department of Biomedical Sciences, Faculty of Health Sciences International Hellenic University Thessaloniki Greece

**Keywords:** cervical insufficiency, collagen, gene, hereditary thrombophilia mutations, inflammation, polymorphism, pregnancy loss, preterm birth, progesterone

## Abstract

Cervical insufficiency (CI) is characterized by spontaneous dilation of the cervix in the absence of painful uterine contractions in the mid‐trimester, leading to premature delivery. It is responsible for up to 20% of second trimester pregnancy losses, mostly <24 weeks. This life‐threatening condition for the growing fetus faced during pregnancy is of complex etiology. The genetic background of this disorder is of fundamental importance. A number of genes have been implicated in CI, highlighting the role of collagen, inflammation, immune, and coagulative factors as key players in CI pathogenesis. This review sheds light on current knowledge regarding the genetic component of CI, analyzing the genes that have been associated with this pathology and describing already identified pathways that participate in CI pathogenesis. Current scientific gaps and future research directions are also discussed in an attempt to disentangle the mystery of CI pathophysiology.

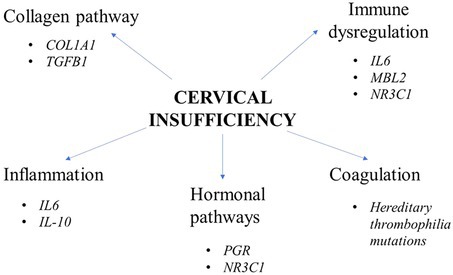

## Graphical abstract

Pathways and genes implicated in CI pathogenesis.

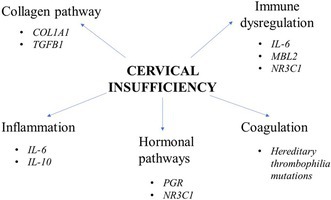

## 1 INTRODUCTION

Cervical insufficiency (CI) is defined as the “the inability of the uterine cervix to retain a pregnancy in the absence of signs and symptoms of clinical contractions, or labor, or both in the second trimester”.^1^ It is potentially followed by prolapse and premature rupture of fetal membranes (PROM), leading to expulsion of a premature fetus and eventually pregnancy loss or preterm birth. The prevalence of CI is approximately 1% of all pregnancies, 20% of second‐trimester pregnancy losses <24 weeks of gestation, as well as 8% of recurrent mid‐trimester pregnancy losses^2^; however, there is a wide variation in the reported CI incidence, probably due to biological differences among different ethnicities or bias between general practitioners and referral centers. Historically, reports of mid‐trimester pregnancy loss due to a primary cervical etiology date back to the 17th century. The term “cervical incompetence” was initially used to describe this condition, which is now more commonly referred as “cervical insufficiency.” The concept of cervical insufficiency was more widely accepted from the middle of the 20th century, and mid‐trimester cerclage procedures were also implemented in clinical practice.^3^

Although there is no standard diagnostic test, painless cervical dilatation on physical examination, an obstetric history of second‐trimester losses or preterm births, or the presence of a short cervix (<25 mm before 24 weeks of gestation) on transvaginal ultrasonography can aid the diagnosis of this pregnancy‐threatening condition. History of cervical surgery, previous miscarriage, or a shortened cervix are common risk factors for CI. However, the exact etiology of this condition is unclear. Cerclage and vaginal progesterone can be considered as possible treatment options.^4^, ^5^ Most guidelines recommend progesterone treatment for cervical shortening, for patients with no history of preterm birth. Those women who have experienced a previous preterm delivery should be offered a cervical cerclage. Regarding multiple gestations, which are also a risk factor of preterm birth, most guidelines recommend against active treatment.^4^

Cervical insufficiency is a complex disease with a substantial genetic basis.^6^, ^7^, ^8^, ^9^ The cervix provides mechanical strength and also acts as a barrier to prevent infection during pregnancy. During pregnancy, the cervix undergoes appropriate remodeling and adaptations to carry out a successful delivery. This remodeling, by which the cervix becomes softer and with increased elasticity can be affected by multiple factors, among which genetic. This review describes several genes that have been associated with CI pathogenesis, discussing the role of genetic polymorphisms in CI, highlighting also the importance of specific pathways in this pregnancy complication (Table 1).

**TABLE 1:** Genetic alterations associated with cervical insufficiency.

Pregnancy disorder	Gene	Gene location	Genetic alteration	Main mechanism	References
Cervical insufficiency	COL1A1	Chr 17q21.33	rs1800012 rs2586490 rs2857396	Abnormal metabolism of collagen	10, 11
	TGFB1	Chr 19q13.2	rs1800471	Abnormal metabolism of collagen	^10^
	IL‐6	Chr 7p15.3	rs2069837	Inflammation, immune dysregulation	12, 13
	IL‐10	Chr 1q31‐q32	IL10.G microsatellite	Inflammation	^14^
	MBL2	Chr 10q11.1–q21	rs5030737 rs1800450 rs1800451 rs11003125 rs7096206 rs7095891	Immune dysregulation	10, 12, 15
	PGR	Chr 11q22‐3	838 G>A 858 C>G 1844G>A 2362C>T 2387C>T 2696G>A	Dysregulated hormonal pathway	^16^
	NR3C1	Chr 5	1084G>A 1475A>G	Dysregulated hormonal pathway, immune dysregulation	^16^
	Hereditary thrombophilia	Chr 1q23 Chr 11p11‐q12	FVL 1691G>A 20210G>A	Increased coagulation	^17^

Abbreviations: COL1A1, Collagen Type I Alpha 1 Chain; IL‐10, Interleukin 10; IL6, Interleukin‐6; MBL2, Mannose‐binding lectin; NR3C1, Nuclear Receptor Subfamily 3 Group C Member 1; PGR, Progesterone receptor; TGFB1, Transforming growth factor beta 1.

## 2 METHODS

For this narrative review article, we searched PubMed and Scopus databases for peer‐reviewed research, review articles, and meta‐analyses regarding the role of genetics in CI, with no time restrictions. The keywords used were gene, polymorphism, genetic, susceptibility, risk factor, familial cases, cervical insufficiency, preterm birth, recurrent pregnancy loss, allele, mutation, and genotype, in various combinations. We also screened the references of the selected articles for possible additional articles i to include most of the key recent evidence. Regarding the inclusion criteria, all studies were in the English language, performed on humans, and referred to genetics in women with CI. Studies were excluded when the title and/or the abstract were not compatible with the aim of this narrative review.

## 3 RESULTS AND DISCUSSION

### 3.1 COL1A1

Collagen is an extracellular matrix protein that constitutes approximately 85% of the dry weight of the cervix. As pregnancy progresses, there is a remodeling of the cervix including collagen degradation, promoting cervical softness and elasticity.^18^ Type I and III collagens are the basic collagens of the cervix. The type I collagen consists of one alpha 2 and two alpha 1 chains, which are encoded by the collagen type I alpha 2 chain (*COL1A2*) and collagen type I alpha 1 chain (*COL1A1*) genes, respectively. Type III collagen consists of three alpha‐1 chains.

The collagen type I alpha 1 chain (*COL1A1*) gene is located on chromosome 17q21.33 and consists of 51 exons. It is present in most connective tissues and in bone, cornea, dermis, and tendon.^19^, ^20^ Mutations in this gene have been associated with connective tissue diseases like osteogenesis imperfecta, Ehlers–Danlos syndrome, and Caffey disease.^21^ There is also substantial evidence supporting that collagen dysregulation can play a crucial role in CI, as decreased concentration of collagen, hyaluronic acid, and sulfated glycosaminoglycans and an increase in collagen extractability after labor have been observed in the cervixes of pregnant versus nonpregnant women.^22^, ^23^ Moreover, decreased levels of hydroxyproline have also been documented in non‐pregnant women with a history of CI,^24^ suggesting that connective tissue dynamics are important for the proper function of the cervix.

*COL1A1* has a collagen transcription regulation region in its first intron, which is called the Sp1 transcription factor binding site. The impact of *COL1A1* polymorphisms in CI susceptibility has been examined, with increased interest regarding the rs1800012 *COL1A1* polymorphism, which has been found to affect collagen expression. More specifically, rs1800012 is characterized by the substitution of a guanidine (G) to a thymidine (T) residue (G → T). This alteration is associated with increased binding affinity to the Sp1 transcription factor, affecting COL1A1 levels and probably the mechanical strength of the cervix, thus decreasing the risk of CI.

Studies have examined the *COL1A1* rs1800012 polymorphism in CI, highlighting the importance of investigating the role of specific genetic polymorphisms in different ethnicities.^6^, ^12^ Another polymorphism, rs2586490, has also been associated with CI.^11^ The homozygote mutant genotype was found to have a higher frequency in the CI group compared to controls. The rs2586490 variant is located in a transcription factor binding site and is recognized by twist basic helix–loop–helix transcription factor 1 (TWIST1), affecting transcription. TWIST1 has been found, among others, to affect the expression of proinflammatory cytokines, including tumor necrosis factor alpha and interleukin 1 beta (IL1B). Moreover, the rs2857396 *COL1A1* polymorphism has been shown to have a protective effect for the gestational age of delivery in women with CI.^11^ Interestingly, Gulucu et al. reported a significant difference in the genotype frequencies of *COL1A1* rs1800012 (G > T) polymorphism in pregnant women with CI compared to healthy controls. The TT genotype, which has been associated with increased production of pro‐alpha1 chains of type I collagen, exerting a protective role against CI, was approximately seven times more common in the control compared to the patient group. The *COL1A1* rs1800012 polymorphism was also found to be associated with the history of cerclage.^10^ These results concerning *COL1A1* polymorphisms need to be replicated across diverse populations. The importance of genetic variability in *COL1A1* regarding CI is also demonstrated in a study by Shah et al. in which bioinformatic analysis identified additional genes and pathways linked to *COL1A1* and CI pathogenesis.^25^ COL1A1 associated gene cluster protein–protein interaction assessment shows that COL1A1 gene protein is strongly connected with the ADAMTS2 (ADAM metallopeptidase with thrombospondin type 1 motif 2), PCOLC (procollagen C‐endopeptidase enhancer), SPARC (secreted protein acidic and cysteine‐rich), ITGB1 (integrin subunit beta 1), RUNX2 (RUNX family transcription factor 2), and other collagen family proteins like COL1A2 (collagen alpha‐2 type I), COL3A1 (collagen alpha‐3 type I), out of which RUNX2 shows a lower level of interaction as compared to other genes.^25^

### 3.2 TGFB1

The transforming growth factor beta 1 (*TGFB1*) gene is located on chromosome 19q13.2 and has seven exons.^26^ It encodes for a multifunctional peptide that belongs to the profibrotic cytokine family and participates in processes like differentiation, proliferation, and angiogenesis, as well as the synthesis and deposition of extracellular matrix.^27^, ^28^ Additionally, it is a pivotal regulator of collagen synthesis and a crucial player in the attachment of trophoblast cells to the extracellular matrix, in embryo implantation, as well as in the preservation of a healthy, uncomplicated pregnancy.^29^ Moreover, most cells in the human body like epithelial, endothelial, hematopoietic, and cells of the connective tissue, have *TGFB1* receptors, highlighting the fundamental role of TGFB1 pathways in the human body, especially in the extracellular matrix.

The rs1800471 (also designated as +915 G > C, c. + 74 G > C, and Arg25Pro) polymorphism in exon 1 of the *TGF‐*B*1* gene has gained particular interest, as it interferes in the transport of the encoded protein across the endoplasmic reticulum membrane, regulating TGFB1 levels.^30^ Moreover, according to in silico analysis, the *TGFB1* rs1800471 polymorphism significantly modified the secondary mRNA structure, reducing mRNA half‐life and affecting TGFB1 levels.^31^ In addition, this polymorphism made the TGFB1 molecule more hydrophobic, inhibiting TGFB1 transport across the endoplasmic reticulum membrane. Gulucu et al. recently found significant differences in the genotype and allele frequencies of the *TGFB1* rs1800471 (G > C) polymorphism between the CI and control group.^10^ More specifically, the C allele and the C allele‐carrying genotypes (GC + CC) were significantly overrepresented in patients compared to controls, increasing susceptibility to CI.^10^ This result remains to be replicated among various ethnicities. Interestingly, in the study of Gulucu et al., except for the *TGFB1* rs1800471 polymorphism, the *COL1A1* rs1800012 variant was also examined; the combined frequency of the TT/GG genotypes of *COL1A1* rs1800012/*TGFB1* rs1800471 polymorphisms was observed to be significantly lower in the CI group than in controls, suggesting a protective role of the TT genotype against CI (17). Functional analysis regarding the effect of *TGFB1* and *COL1A1* gene alterations on mRNA and protein expression would be essential for better understanding of the role of these genes in CI.

### 3.3 IL‐6

The interleukin‐6 (*IL‐6*) gene is located on chromosome 7p15.3. It includes four introns and five exons and it encodes a small polypeptide (19–28 kDa) with a pleiotropic effect on inflammation, immune response, and hematopoiesis.^32^ IL‐6 is produced by a number of cells, including B and T lymphocytes, macrophages, fibroblasts, keratinocytes, vascular endothelial cells, and dendritic cells.^33^ Its production is triggered in response to infections and tissue injuries. Dysregulation of IL‐6 has been found to be implicated in a number of inflammatory cascades underlying the pathophysiology of multiple pregnancy complications^34^; thus, the role of this pro‐inflammatory cytokine has been examined in CI as well.

Increased levels of amniotic fluid IL‐6 in women with CI undergoing amniocentesis compared to controls have been observed, too.^35^ Interestingly, in a recent study, the levels of inflammatory factors, such as cytokines, were measured in women with CI and preterm birth. Both in endocervical and exocervical samples, IL‐6 was significantly increased in the preterm group compared to the term group.^13^ Notably, vaginal levels of IL‐6 have previously been reported to be predictive in patients with CI,^36^, ^37^ supporting a possible role of this cytokine in CI. In a case–control study, 30 women with a history of second‐trimester miscarriage or preterm birth due to CI and 70 controls were examined. Homozygous carriers of the IL6‐174 genotype GG (rs2069837) had an odds ratio (OR) of 3.1 for CI compared to controls, suggesting that *IL‐6* polymorphisms might increase susceptibility to preterm birth due to CI.^12^ Larger studies in different populations are awaited to elucidate the exact role of the rs2069837 polymorphism and its association with CI and/ or preterm birth.

### 3.4 IL‐10

The interleukin 10 (*IL‐10*) gene is located on chromosome 1q31–q32.^38^ It encodes for a pleiotropic cytokine with a critical role in regulating inflammation and in maintaining cell homeostasis; it mainly acts as an anti‐inflammatory cytokine that can downregulate the synthesis of proinflammatory cytokines and inhibit Th1 type immune response. Cytokines and increased inflammation have been found to be implicated in CI pathogenesis.^14^

In a study by Warren et al., the IL10.G13 allele in the microsatellite of the promoter region of *IL‐10* was overrepresented in women with CI compared to controls, suggesting that alterations in the maternal inflammatory environment might increase susceptibility to CI. The results need to be replicated in different ethnic groups. Interestingly, this highly polymorphic IL10.G microsatellite in the IL‐10 promoter region has been described to decrease IL‐10 levels.^14^ Additional studies regarding the role of IL‐10 and other cytokines in CI have to be carried out to elucidate the role of inflammation in CI.

### 3.5 MBL2

The mannose‐binding lectin (*MBL2*) gene is located on the chromosome 10q11.1–q21 and it encodes for MBL protein, which belongs to the collectin family and has multiple antimicrobial activities.^39^ As a “pattern recognition molecule” it recognizes various pathogens such as viruses, bacteria, fungi, or parasites, subsequently activating the complement system or the phagocytosis of the microorganisms by macrophages. Thus, MBL2 is considered a first‐line immune defense against infections.^15^

MBL is produced in the liver and its plasma levels are determined genetically by three polymorphisms in exon 1 of *MBL2* and three polymorphisms in the *MBL2* promoter region. In a study by Sundtoft et al., *MBL2* genotypes coding for low or intermediate levels of plasma MBL had an OR of 3.3 for CI compared to controls.^24^ Moreover, serum MBL levels were decreased in women with CI compared to controls. Thus, *MBL2* and its low plasma levels might increase susceptibility to preterm birth associated with CI.^12^ Similarly, a trend for low‐producing *MBL2* genotypes was observed in women with recurrent late pregnancy losses with a clinical picture resembling CI.^40^ Larger studies and in different ethnic groups are required to draw more definite conclusions.

### 3.6 PGR

Progesterone (PG) is produced by the corpus luteum in the ovaries and is a fundamental steroid hormone necessary for the preparation of the uterus for pregnancy, as well as for the maintenance of pregnancy.^41^ PG is also crucial for retaining cervical integrity before labor induction and has been found to prevent cervical collagen decomposition.^42^, ^43^ The onset of labor has been connected with withdrawal of PG, whereas PG supplementation has been used for CI and PTB prevention in high‐risk women.^44^ PG acts via binding to progesterone receptors (PGR) PGR‐A and PGR‐B, which are both transcribed from a single gene located on chromosome 11q22‐3. Both cervical stromal and epithelial cells express PG receptors.^45^

In Voložonoka et al., statistically significant overrepresentation of rare damaging *PGR* variants was observed in CI women compared to the control group, suggesting a hormonal implication in the pathophysiology of CI.^16^ In silico and three‐dimensional analysis of these genetic alterations showed that three of them, one located within the DNA binding domain and two in the hormone/ligand binding domain, were pathogenic, probably leading to early PG withdrawal or affecting the effectiveness of exogenous PG supplementation.^16^ Some limitations of the study were the relatively modest sample size, the absence of a matched control group and a bias due to targeted selection of individuals included in the study. Dysregulated progesterone action pathways could have a pivotal role in CI pathogenesis; however, how hormonal pathways participate in CI pathophysiology remains to be further investigated.

### 3.7 NR3C1

Nuclear receptor subfamily 3 group C member 1 (*NR3C1*) gene is located on chromosome 5. It consists of 17 exons, nine of which are non‐coding exons in the promoter of the gene. *NR3C1* encodes for the glucocorticoid receptor where cortisol can bind to and exert multiple functions, including anti‐inflammatory and immunosuppressive effects. Interestingly, PG can also bind to this receptor.^46^

Genetic alterations in *NR3C1* have been suggested to dysregulate immune system tolerance during pregnancy increasing the risk of CI and/or PTB. In Voložonoka et al., rare damaging variants in *NR3C1* were identified within key functional regions, probably affecting the receptors' DNA binding capability.^16^ Larger well‐designed studies are warranted to draw more definite conclusions.

### 3.8 Hereditary thrombophilia mutations

Pregnancy is characterized by a hypercoagulable state that in some situations can lead to thrombotic events of the uteroplacental circulation and adverse pregnancy complications. Hypercoagulation can be triggered by inflammatory processes, and thrombin is known to be a key player in these processes. In general, inflammation upregulate procoagulant factors downregulate natural anticoagulants and inhibit fibrinolytic activity.^47^

Hereditary thrombophilia is associated with two or more gene defects,^48^ mainly Factor V Leiden (FVL) and prothrombin G20210A mutations. FVL results in a mutated variant of factor V, which increases resistance to inactivation by protein C and the risk of thrombosis by 5‐to‐10‐fold in heterozygotes and by 80‐fold in homozygotes.^49^, ^50^ The prothrombin G20210A mutation also results in increased prothrombin levels. Ulander et al. found that hereditary thrombophilia FVL and G20210A were overrepresented in CI women compared to controls, suggesting that hereditary thrombophilia could be a risk factor for CI.^17^ However, this finding needs further replication.^12^

### 3.9 Research gaps and future directions

Although current research on CI is intense, several issues have to be addressed to better understand this condition. Much of the available data is based on animal models; as similarities and differences exist between species, future research should focus on human tissue studies to better translate findings to human physiology. Except for limited human data, the lack of consistent definitions, as well as research methodologies, raise difficulties in elucidating CI pathophysiology. Moreover, many studies depend on results obtained from postpartum cervical biopsies, mainly examining the tissue compared to the active remodeling phase.

The number of genes identified as candidate susceptibility factors of CI is also limited. Based on current knowledge, the pathways and mechanisms that have been implicated in CI include inflammation, perturbations in collagen homeostasis, dysregulation in hormonal pathways, immune dysfunction, as well as increased coagulation. Interestingly, Son et al.^51^ showed that women with CI exhibit a differential transcriptomic profile compared to controls. In fact, 30 genes were differentially expressed between CI and controls, among which neutrophil‐mediated immunity‐associated (DEFA3 and ELANE) and bicarbonate transport‐related genes were overexpressed in the CI group. Serum levels of alpha defensin 3 were significantly overrepresented in CI women, as well. Moreover, immune and defense response to organism‐associated genes and influenza A and NOD‐like receptor signaling pathways were also upregulated in the CI‐term group, pinpointing additional pathways and genetic factors contributing to CI. Thus, there is currently an intriguing challenge to identify novel pathways and genes contributing to CI. Careful patient selection is fundamental in this effort, as well as the examination of larger sample sizes to increase statistical power. Paying attention to family histories will allow us to identify women at increased risk for CI. Genetic studies in different populations are also awaited to increase our knowledge regarding the role of genetic polymorphisms in CI in different ethnicities. Whole exome, whole genome, and functional studies are also needed to identify new potential causative genes. Well‐matched control groups should also be used in future studies. Identifying markers for premature cervical remodeling is undoubtedly essential for developing effective screening methods. Assessing clinical, non‐invasive imaging data and genetic factors will significantly increase our understanding regarding cervical remodeling. Artificial intelligence might also aid the recognition of women at increased risk of CI, and novel strategies are awaited to be developed for the prompt and effective treatment of CI based on risk stratification. Cellular mechanisms during cervical remodeling should be also investigated in samples of cervical tissue aiming to acheive early recognition of cervical changes in high‐risk women during pregnancy and the identification of novel biomarkers. The role of lifestyle, maternal health status, and environmental factors on cervical modifications should also be examined. Finally, the impact of gene–gene and gene–environment interactions, as well as of epigenetic changes should also be thoroughly studied in an attempt to disentangle the mystery of this complex and heterogeneous disorder.

## 4 CONCLUSION

Cervical insufficiency is a disease of complex etiology that is currently largely unidentified. The genetic component of CI is supported by the increased incidence of familial cases with this disorder and is further reinforced by remarkable data indicating specific genetic risk factors for CI. All this information enhances the need for further investigation of the genetic background of this complex disorder to discover novel mechanisms and pathways that participate in CI pathogenesis. According to current knowledge, collagen homeostasis, inflammation, immune dysfunction, and exacerbated coagulation are important factors in CI. However, these seem to be only the “tip of the iceberg”; additional studies are needed to better understand CI pathophysiology and follow cause‐specific interventions in this stressful pregnancy disorder.

## AUTHOR CONTRIBUTIONS

Kallirhoe Kalinderi had the idea for the article, Kallirhoe Kalinderi and Michail Kalinderis performed the literature search and analysis and drafted the manuscript. Maria Chatzidimitriou and Liana Fidani critically revised the work. All the authors read and approved the final manuscript for submission.

## FUNDING INFORMATION

This research received no external funding.

## CONFLICT OF INTEREST STATEMENT

The authors have no conflicts of interest.

## Data Availability

No new data were created or analyzed in this study. Data sharing is not applicable to this article.
